# Involvement and Clinical Aspects of MicroRNA in Osteosarcoma

**DOI:** 10.3390/ijms17060877

**Published:** 2016-06-03

**Authors:** Ram Mohan Ram Kumar, Aleksandar Boro, Bruno Fuchs

**Affiliations:** 1 Department of Experimental Pathology, CHUV and University of Lausanne, 1011 Lausanne, Switzerland; 2 Laboratory for Orthopaedic Research, Department of Orthopaedics, Balgrist University Hospital, University of Zurich, 8008 Zurich, Switzerland; aboro@research.balgrist.ch (A.B.); bruno.fuchs@balgrist.ch (B.F.)

**Keywords:** osteosarcoma, metastasis, microRNA, biomarker, cancer

## Abstract

Osteosarcoma (OS) is the most common primary bone cancer in children and adolescents, but its pathogenesis has been difficult to establish because of its well-known heterogeneous nature. OS has been associated with genetic and cytogenetic abnormalities, which include function-impairing mutations in tumor suppressors and the activation of oncogenes. OS tumorigenesis has been linked to alterations of several genes characterized by a high level of genetic instability and recurrent DNA amplifications and deletions. MicroRNAs (miRNAs), 18–25-nucleotide noncoding RNAs, are critical for various biological processes like differentiation, cell growth and cell death. Dysregulation of miRNA expression leads to phenotypic and genotypic changes in cells, which leads to cancer. Studies on miRNAs have initiated a significant effect in both diagnosis and treatment of cancer. This review focuses on the current knowledge of clinical applications of miRNAs for the better diagnosis and management of OS.

## 1. Introduction

Despite technological advances in diagnosis and treatment of osteosarcoma (OS), the mortality rate remains high. The survival rate is estimated to be around 60%–80% in patients treated with multidrug chemotherapy and local control interventions [1]. There is an urgent need to develop a more effective therapy than the conventional ones for the treatment of OS. Translational studies using miRNA have gained a lot of interest, ranging from those on basic pathogenesis to those on clinical applications. Recently, studies have demonstrated the involvement of miRNAs in cancer progression and metastasis. In this review, we outline the current status on how miRNA research has progressed in OS recently, and how useful miRNAs are in clinical practice

## 2. MicroRNAs

MicroRNAs (miRNAs), single-stranded non-coding RNA molecules, contain 18–25 nucleotides, and mediate the expression of various eukaryotic genes [2]. miRNAs are involved in various biological processes that regulate differentiation, apoptosis and proliferation by complementarily pairing with 3′ untranslated region (3′ UTR) of target genes [3,4], thereby inhibiting mRNA translation of those genes. It has been known that a single miRNA can affect multiple mRNAs or multiple miRNAs may affect single mRNAs [2]. There is a widely accepted hypothesis of miRNA function, but the mechanistic function of miRNA biogenesis and gene silencing are still not properly understood. In a recent review, the nuclear events in the miRNA biogenesis pathway have been explained in detail [5].

## 3. miRNAs and Cancer

In humans, miRNA genes are localized within the genomic fragile sites and are frequently subjected to phenotypic and genotypic changes in the cell, which lead to cancer [2]. Most of the distinct miRNAs studied are commonly up or downregulated in distinct types of human neoplasia and are often being associated with distinct cytogenetic abnormalities. Expression profiles of miRNAs are known to be altered in many tumors such as chronic lymphocytic leukemia (CLL) [6], acute lymphoblastic leukemia (ALL) [7], pancreatic cancer [8], *etc.*, implying that miRNA may be involved in the development of cancer and other diseases. In cancer, a unique feature of miRNA expression is that each tumor tissue contains specific miRNA signatures that are distinctly different from normal tissues. Based on this difference, cancers can be subclassified into prognostic groups according to their miRNA signatures [9,10]. The role of miRNA in cancer has been widely explored as studies have revealed mutual interactions between miRNA and previously known tumor initiating genes [2].

## 4. miRNA and Osteosarcoma

The mechanism of action of miRNAs in OS is not understood clearly. Only some studies could explain the role of miRNAs in OS pathogenesis. miRNAs are known to function as both oncogenes as well as tumor suppressors in OS and influence the phenotypic characteristic of OS cells via regulation of their target genes [11]. Table 1 lists the functions of miRNA and gene expression profiles and their respective target genes in OS. Recently, miR-27a has been found to be elevated in sera of OS patients, which could effectively differentiate from the healthy controls and could be used as a diagnostic marker [12]. In another study, miR-130b was found to be elevated in OS tissues that showed a strong correlation with the aggressive progression of OS, affecting overall survival [13]. Jones *et al*. reported unique OS signatures of miRNA expression related to pathogenesis and clinical metastasis. Expression of miR-181b, miR-29b, miR-16 and miR-142-5p were found to be downregulated in subpopulations of OS tumor cells. Furthermore, higher expression of miR-181a, miR-181b, miR-181c and miR-27a were highly expressed in biopsy samples in patients who developed clinically metastatic disease. In spite of a small sample size in their study, the conventional OS miRNA expression signature showed strong statistical significance indicating the possibility that these miRNAs play a central role in osteosarcomagenesis [14]. Poos *et al*. investigated the proliferation effects of OS cells by studying the interplay between miRNAs and the transcription factor (TF) co-regulatory network. The database of the regulatory network between miRNAs and TFs recognized in their analysis are available online (http://www.complex-systems.uni-muenster.de/co_networks.html). In their study, low expression of miR-9-5p, miR-138, and miR-214 resulted in a strong proliferative phenotype of OS cells due to their impact on TFs, NFKB and RB1. Additionally, they also identified miR-9-5p, miR-138, and miR-214 interacting with TFs, SP1 and MYC leading to a high proliferative phenotype of OS cell lines [15].

Proliferation, invasion, and metastasis of OS tumor cells are also influenced by miRNA expression. Cao *et al.* reported upregulation of miR-802 in OS tumor tissue and induced proliferation by regulating p27 expression, thereby classifying miR-802 as an onco-miRNA [16]. Since OS is characterized by a high propensity for lung metastasis in patients leading to death, some studies have reported the anti-metastatic effects of miRNA in OS. Over expression of miR-143 in 143B cell lines regulated MMP-13 expression levels, and the invasion of cells was significantly reduced. Intravenous injection of miR-143 in mice significantly suppressed lung metastasis derived from 143B cells [17]. Recently, studies of miR-194 overexpression in U2OS and SOSP-9607 cells resulted in reduced proliferation, migration, and invasion of OS cells *in vitro* and significantly reduced tumor growth and pulmonary metastasis *in vivo* [18]. Table 2 lists the miRNAs that are reported to have anti-metastatic effects in OS.

The malignant phenotype of OS might not be the result of dysregulation of miRNA alone, while comprehensive analysis of miRNA would provide more insights into the molecular mechanisms of OS.

## 5. Circulating miRNA as Biomarkers in Osteosarcoma

Circulating miRNAs are considered as prognostic biomarkers for various types of cancer [39,40]. miRNAs in human serum and plasma exist in remarkably stable forms [41,42], which render the possibility to be used as noninvasive disease markers in cancer. Utilization of plasma miRNA as biomarkers nullifies the collection of tissue samples through invasive procedures like biopsies. Comprehensive screening of plasma miRNA profiles in OS patients would allow early diagnosis of the disease. Yuan *et al.* showed that high serum level of miRNA-21 is correlated to the advanced Enneking stage of tumors and also as a predictive marker for chemotherapeutic resistance and unfavorable prognostic factor for overall survival [43]. In addition to miRNA-21, expressions of miRNA-199a-3p and miRNA-143 were also considered as signature miRNAs to distinguish OS patients from healthy individuals [44]. Plasma levels of miRNA-34b were found to be significantly lower in OS patients compared to individuals without cancer and related with their metastasis status [45]. Zhang *et al.* found that combined elevation of miRNA-196a and miRNA-196b in sera predicted unfavorable prognosis in patients with OS and correlated with tumor grade, metastasis status and recurrence [46]. Circulating miRNA-195, miRNA-133b, miRNA-206 and the miRNA-29 family were all shown to correlate with tumor grade, metastasis overall, and disease free survival in OS patients [47,48,49]. Most importantly, miRNA-9 and miRNA-148a were found to be elevated in serum of OS patients and their levels correlated to tumor size in addition to other clinical parameters [50,51]. Rhoades *et al.* determined the diagnostic application of miRNA-205-5p, miRNA-574-3p, miRNA-335-5p and miRNA-214 as plasma biomarkers for OS, with emphasis on miRNA-214 as an independent marker for metastasis and overall survival in patients [52]. Recently, Lian *et al*. determined that miRNA-195-5p, miRNA-199a-3p, miRNA-320a and miRNA-374a-5p are all upregulated in plasma of OS patients in comparison to healthy individuals, having a strong diagnostic power as a combined marker. Moreover, circulating miRNA-195-5p and miRNA-199a-3p was also correlated with metastasis status, while miRNA-320a and miRNA-199a-3p were correlated with histological subtype [53]. 

Despite the enormous clinical potential of miRNAs as circulating biomarkers in OS as presented in this review, there are several limitations that must be acknowledged. In most of the studies, the cohort of patients with OS was relatively small, and, therefore, experiments with long-term, controlled and large sample sizes are required. Furthermore, the lack of a standardized approach in methodology of normalization of circulating miRNAs is also concerning. Therefore, a considerate approach is required in future studies to establish miRNAs as circulating biomarkers for OS into the clinics.

## 6. miRNA-Based Therapies in Osteosarcoma

The importance and involvement of miRNA function in the progression of OS has paved the way for utilizing miRNAs a possible novel therapeutic targets. The strategic approach involves the use of oligonucleotides or anti-viral constructs to block the expression of an oncomiR or to substitute for the loss of expression of a tumor suppressor miRNA using miRNA mimics [54]. A therapeutic approach by intravenous injection of miR-143 resulted in a significant reduction of lung metastases in eight of the ten mice [17]. Even though miR-143 is effective against pulmonary metastasis, much needs to be studied regarding whether it could be used as target for OS therapy in patients. Recently, Xu *et al*. identified that expression levels of miR-382 were significantly downregulated in highly proliferative OS cell lines and relapsed OS tissue samples compared to their non-invasive cell lines and primary OS samples, respectively. Their clinical data analysis showed that miR-382 expression level was positively associated with metastasis-free survival and inversely associated with relapse in OS patients. This suggests that miR-382 negatively regulates OS cell metastasis and relapse. Combination therapy of miR-382 with conventional chemotherapy prevented OS relapse in their xenograft mouse models. Overexpression of miR-382 in invasive OS cell lines significantly inhibited migration and invasion *in vitro*, while inhibition of miR-382 significantly increased cell invasion and migration. Stable overexpression of miR-382 suppressed the formation of lung metastatic lesions as well as decreased the cancer stem cell (CSC) population and function in OS cells [55]. miR-382 therefore appears to be a promising target in the treatment of OS metastasis. Yuan *et al*. identified that the expression levels of miR-451 were downregulated in OS cell lines and primary tumor samples. A significant correlation between low miR-451 expression and clinicopathological features in OS patients were found out to have shorter disease-free survival [56]. miR-451 seems to feature as a potential novel target for gene therapy of OS.

miRNA dysregulation has been associated with chemotherapy and drug resistance in a variety of cancers like head and neck squamous cell carcinoma (HNSCC), as well as cancers of the breast, *etc.* [57,58]. However, the mechanisms of chemo and drug resistance due to miRNA activation in OS are not well understood. Studies are required to analyze high throughput miRNA expression analysis to identify miRNAs associated with chemo and drug resistance in OS. Recently, Zhang *et al.* determined the differential expression of miR-301a on doxorubicin treatment in chemotherapy-resistant OS and chemotherapy-sensitive OS cells. They found that miR-301a was highly expressed in chemotherapy-resistant OS, which indicated that miR-301a is important for chemo resistance of OS. miR-301a promoted HMGCR (HMG-CoA reductase) expression by targeting AMPKα1 (AMP-activated protein kinase alpha 1) and enhanced resistance of OS cells to doxorubicin [59]. 

Even though the role of miRNAs in OS has been studied in detail, it is not clear whether they can be utilized for the treatment of patients with OS. In addition, extensive toxicity studies and preclinical safety would have to be assessed before an miRNA-based therapeutic approach could be considered for patients with OS.

## 7. Conclusions

miRNA-directed gene regulation will pave the way for improving traditional gene therapy approaches in many cancers. Even though novel miRNA pathways and targets in metastatic OS have yet to be determined, it is evident that miRNAs play a role in the progression of OS by regulating proliferation, invasion, adhesion, metastasis, apoptosis and angiogenesis. Studies have determined the complex regulatory role of miRNAs in OS and closely linked them to the clinical outcome of patients. One of the challenges faced is the identification of all targets of miRNAs involved in OS, thereby establishing their contribution to malignancy. The identification and screening of dysregulated miRNAs in patients with OS may help in the development of prognostic biomarkers and for treatment, respectively. Moreover, since therapeutic targeting of miRNAs promises to improve the clinical management of patients with OS, future studies should be able design miRNA-based treatments efficiently with high quality of delivery, therapeutic effects and better safety profiles in animal models before being introduced into the clinics.

## Figures and Tables

**Table 1:** List of miRNAs and their target genes involved in regulation of OS. Arrows indicate up-regulation (↑) or down-regulation of (↓) of expression of miRNAs in osteosarcoma.

miRNA in OS	Function in OS	Expression Levels	Specimen	miRNA Targets in OS	Reference
miR-214	Oncogene	↑	Cell lines and 8 tumour samples	LZTS1	[19]
miR-183	Tumour suppressor	↓	Cell lines and 50 tumour samples	Ezrin	[20]
miR-27a	Oncogene	↑	Cell lines	MAP2K4	[21]
miR-133b	Tumour suppressor	↓	Cell lines and 23 tumour samples	BCL2L2,MCL-1, IGF1R and MET	[22]
miR-199a-3p	Tumour suppressor	↓	Cell lines and 12 tumour samples	–	[23]
miR-124	Tumour suppressor	↓	Cell lines and 70 tumour samples	Rac1	[24]
miR-646	Tumour suppressor	↓	Cell lines and 10 tumour samples	FGF2	[25]
miR-21	Oncogene	↑	Cell lines and tumour samples	RECK	[26]
miR-9	Oncogene	↑	79 Tumour samples	–	[27]
miR-382	Oncogene	↑	Cell lines	MYC	[28]
miR-100	Tumour suppressor	↓	Cell lines	CYR61	[29]
miR-135b	Oncogene	↑	Cell lines and 7 tumour samples	FOXO1	[30]
miR-1/miR133b	Tumour suppressors	↓	Cell lines	–	[31]

**Table 2:** List of miRNAs and their target genes involved in anti-metastatic activity of osteosarcoma.

miRNA in OS	Specimen	Function	miRNA Targets in OS	Reference
miRNA-34a	Cell lines	Inhibit tumor growth and lung metastasis of OS	c-Met	[32]
miRNA-144	Cell lines and 67 tumour samples	Suppresses tumor cell proliferation and metastasis *in vivo*	ROCK1 and ROCK2	[33]
miR-382	34 canine OS tumour and human tumour samples	Suppress metastasis	–	[34]
miRNA-146a	Cell lines and 53 tumour samples	Suppression of cancer cell invasion and metastasis	HAb18G	[35]
miR-195	Cell lines	Inhibit metastasis	CCND1	[36]
miR-133a	Cell lines and 92 tumour samples	Reduces cell viability and promotes cell apoptosis. Suppress lung metastasis	Bcl-xL and Mcl-1	[37]
miRNA-217	Cell lines and 60 tumour samples	Inhibits migration and invasion of OS cells and supress metastasis	WASF3	[38]
